# Supplementary figures and images for: Babies, bugs and brains: How the early microbiome associates with infant brain and behavior development
Source: PLoS One. 2023 Aug 9;18(8):e0288689. doi: 10.1371/journal.pone.0288689 (PMC10411758; doi:10.1371/journal.pone.0288689)

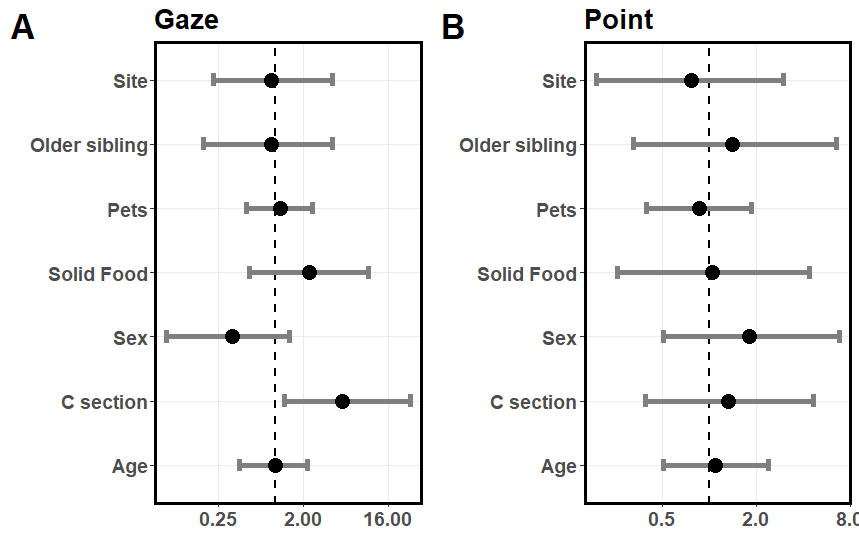

Supplement: S1 Fig — Odds ratio analysis between several metadata variables (collection site, children with older siblings, having pets, children introduced to solid foods, sex (FEM), children mode of delivery and age) and the success on the gaze (A) or point (B) subtest. Only those related to delivery type were significant. (TIF) [file pone.0288689.s001.tif]

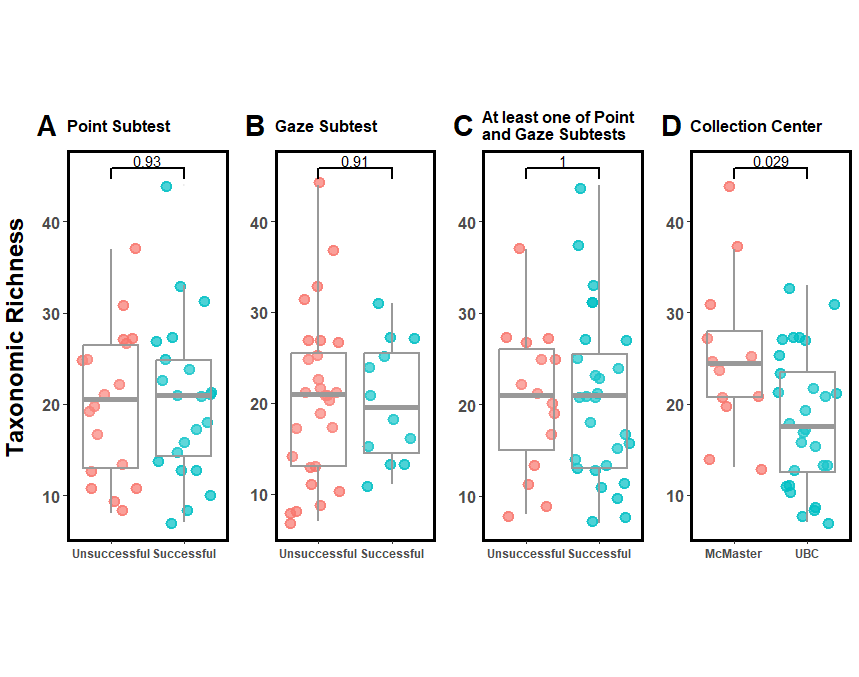

Supplement: S2 Fig — Taxonomic richness analysis comparing those who were unsuccessful versus successful on the point subtest (A), the gaze subtest (B), at least one of the point and gaze subtests (C), as well as comparing the collection sites (D). A two-tailed t-test was performed between the successful and failed attempts. Taxonomic richness had a p-value < 0.05 between collection sites, there was not a statistical difference between the other groups. (TIF) [file pone.0288689.s002.tif]

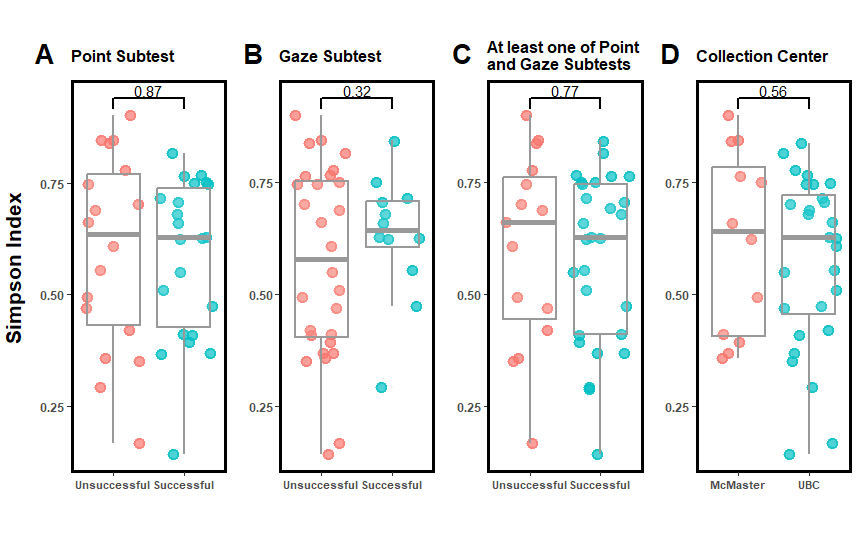

Supplement: S3 Fig — Microbial diversity analysis using Simpson index comparing those who were unsuccessful versus successful on the point subtest (A), the gaze subtest (B), at least one of the point and gaze subtests (C), as well as comparing the collection sites (D). A two-tailed t-test was performed between the successful and failed attempts. There was not a statistical difference between the groups. (TIF) [file pone.0288689.s003.tif]

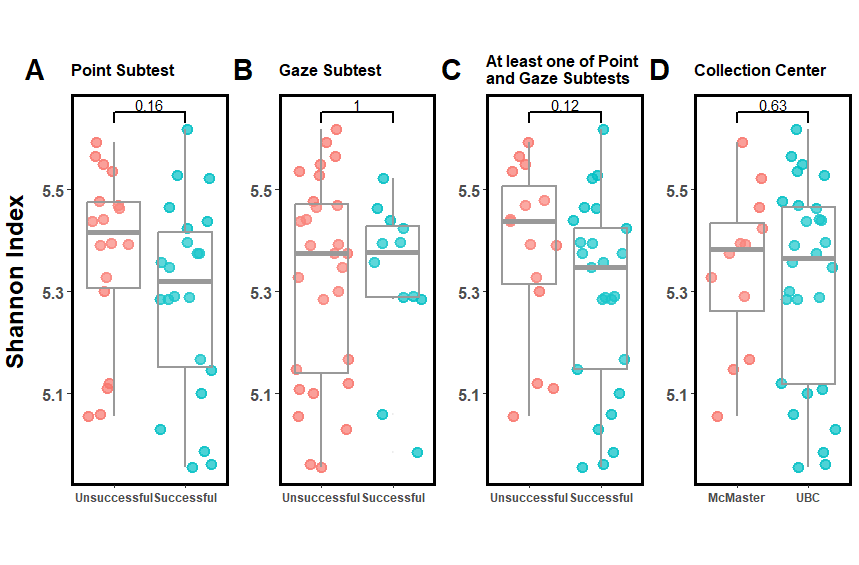

Supplement: S4 Fig — Functional diversity analysis using Shannon index comparing those who were unsuccessful versus successful on the point subtest (A), the gaze subtest (B), at least one of the point and gaze subtests (C), as well as comparing the collection sites (D). A two-tailed t-test was performed between the successful and failed attempts. There was not a statistical difference between the groups. (TIF) [file pone.0288689.s004.tif]

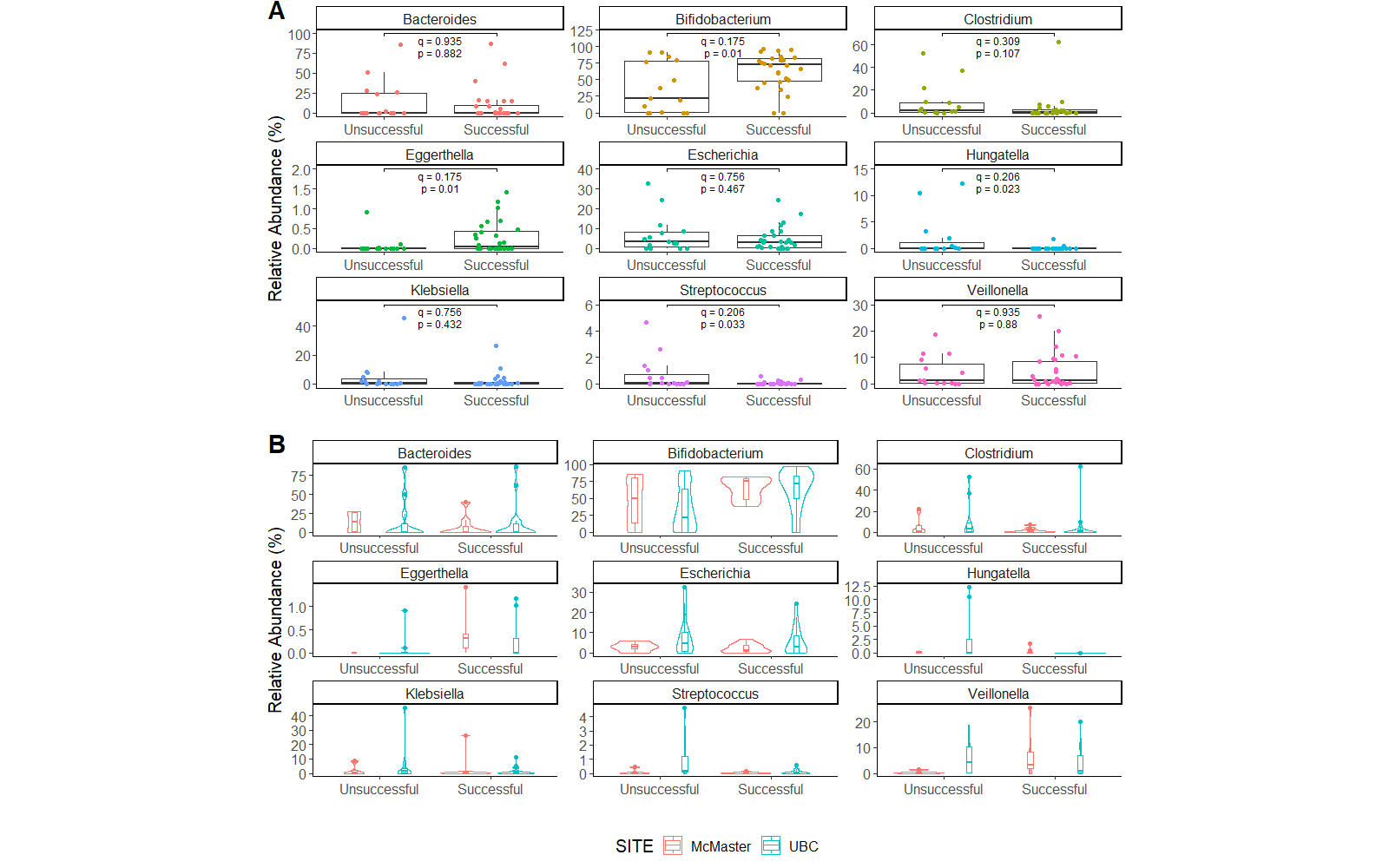

Supplement: S5 Fig — Bacteria relative abundance at the genus level of the most abundant microorganisms in the samples. Difference in bacteria relative abundance by success on at least one of the point and gaze subtests. (TIF) [file pone.0288689.s005.tif]

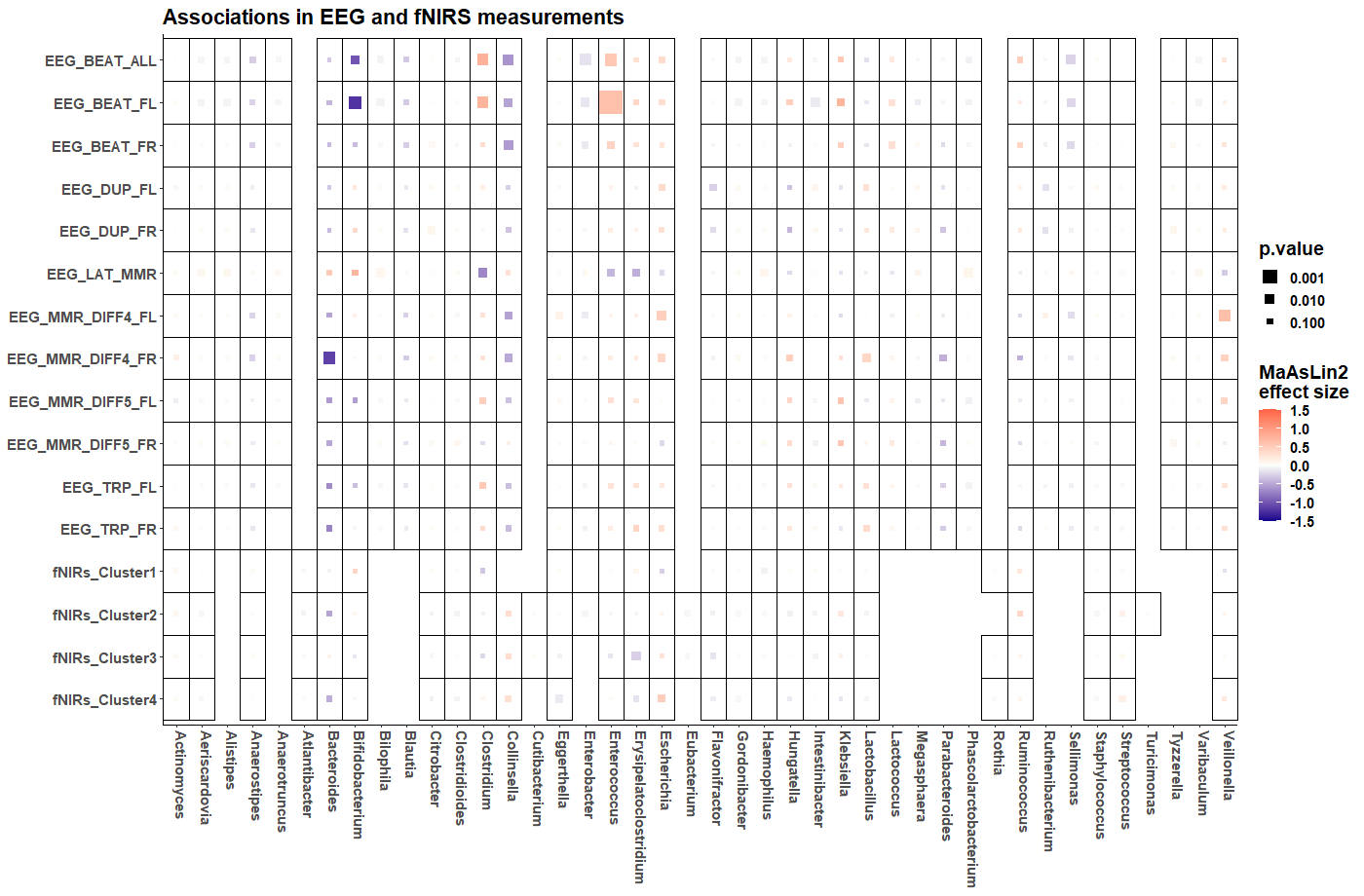

Supplement: S6 Fig — Association analysis between bacteria abundance (CLR transformed) and EEG/fNIRS measurements at the genus level. (TIF) [file pone.0288689.s006.tif]

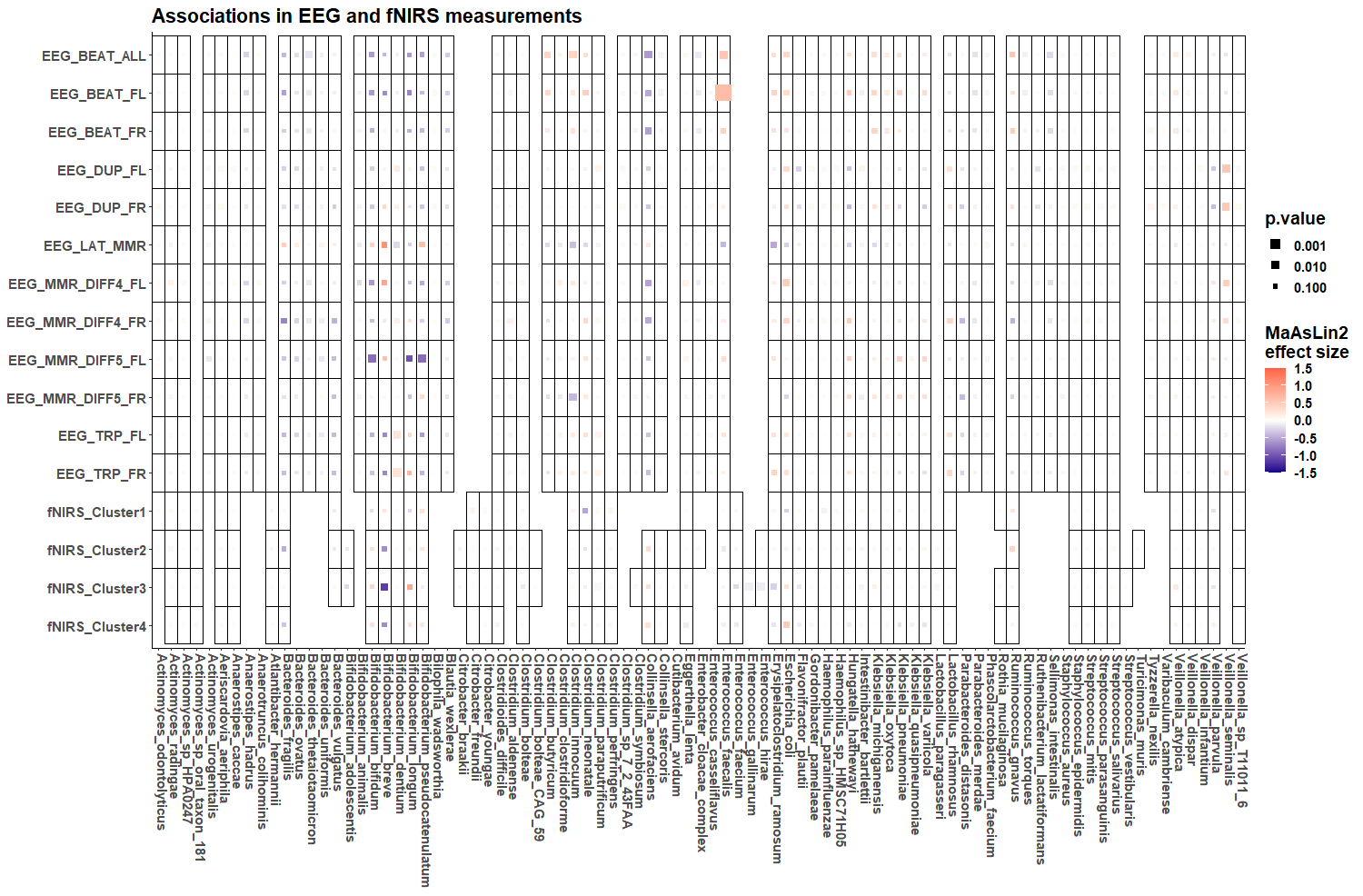

Supplement: S7 Fig — Association analysis between bacteria abundance (CLR transformed) and EEG/fNIRS measurements at the species level. (TIF) [file pone.0288689.s007.tif]

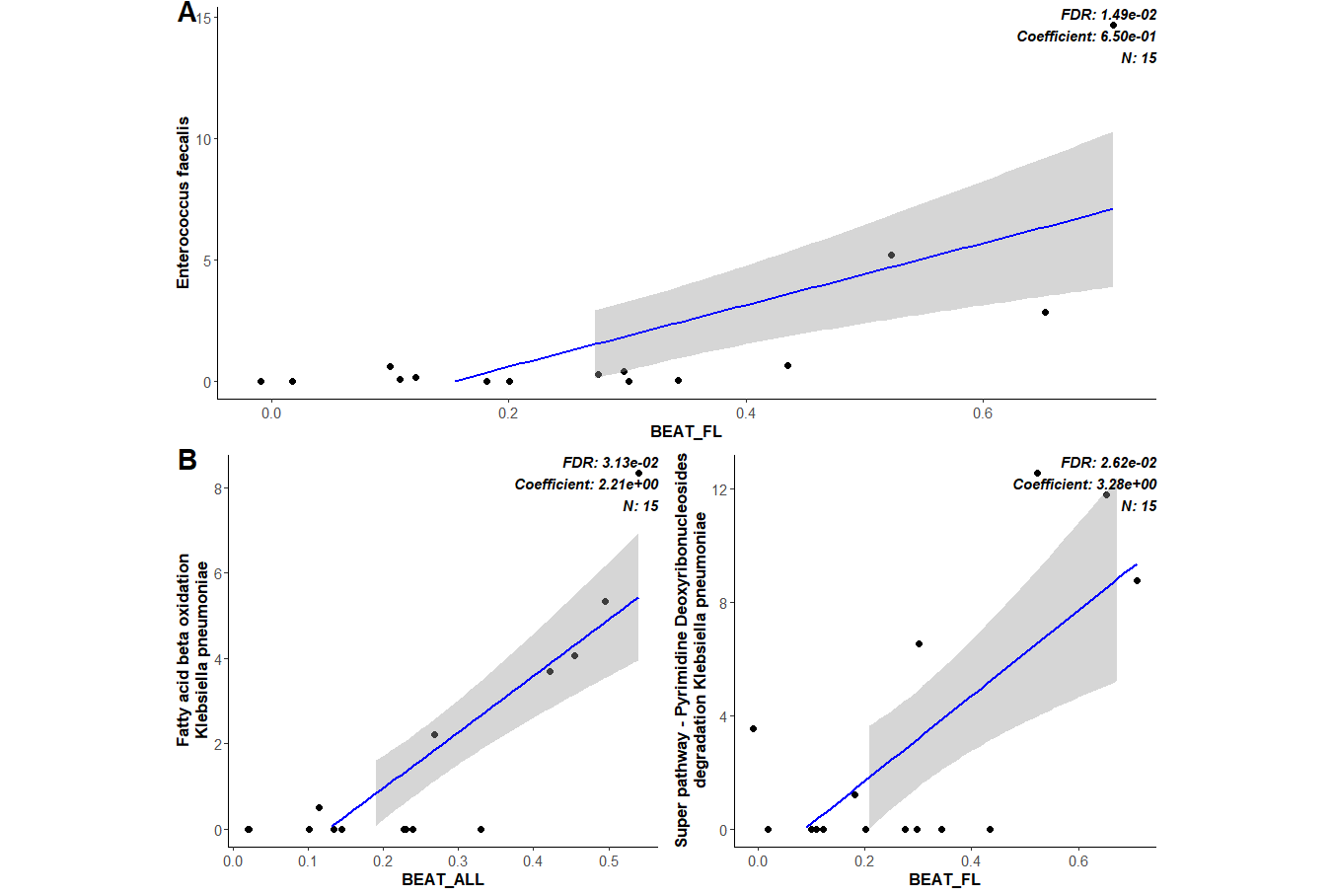

Supplement: S8 Fig — (A) Association analysis between bacteria abundance (CLR transformed) and EEG measurements at the species level. (B) Association analysis between stratified pathways abundances and EEG measurements. Significant associations have an adjusted p-value < 0.05 and a minimum prevalence of 10%. (TIF) [file pone.0288689.s008.tif]

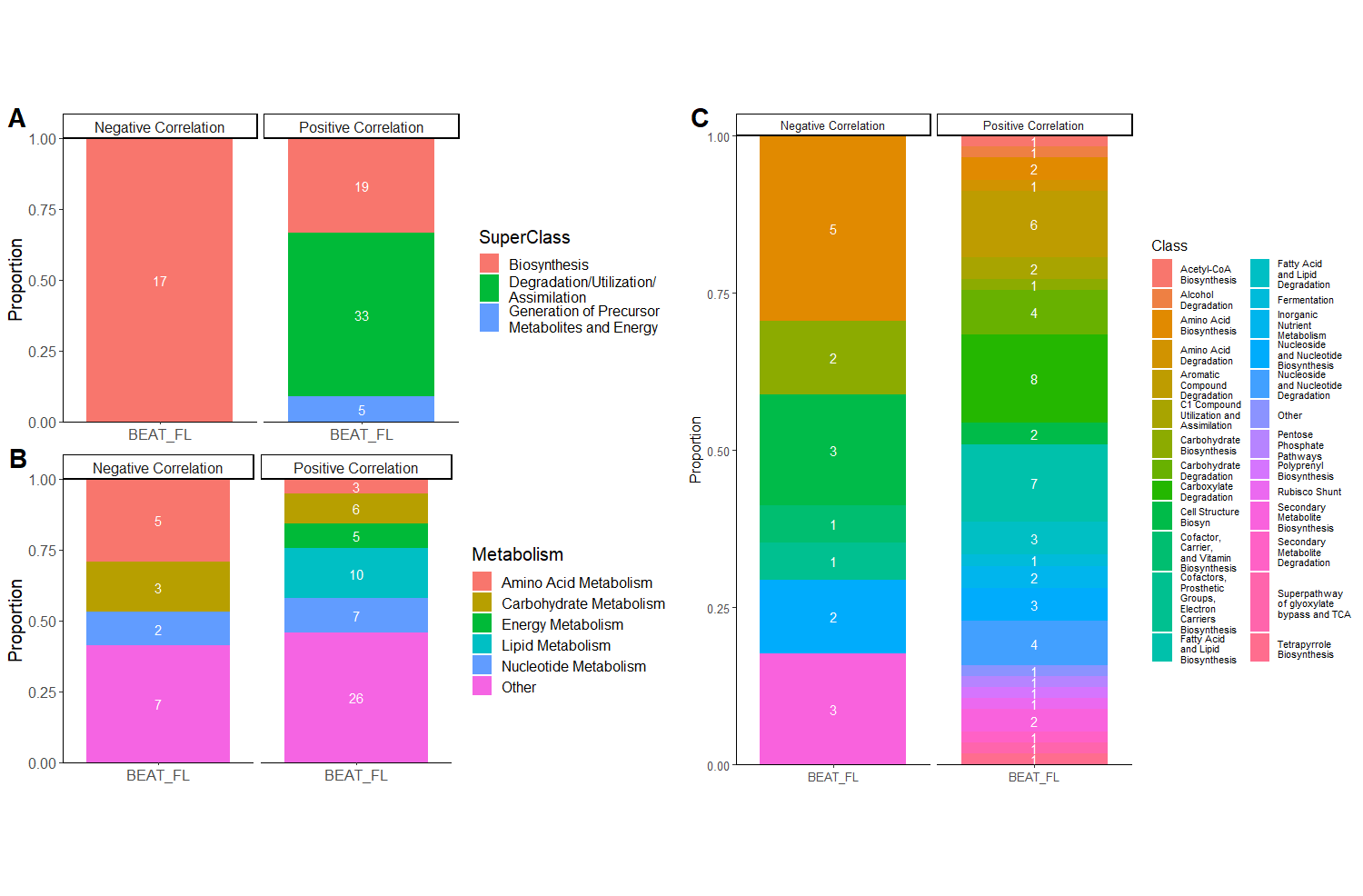

Supplement: S9 Fig — Association analysis between unstratified pathway abundances and EEG measurements. A) Associated pathways at the Super Class level and the variable BEAT_FL faceted by negative or positive associations. B) Association at the metabolism level. C) Association at the Class level. (TIF) [file pone.0288689.s009.tif]

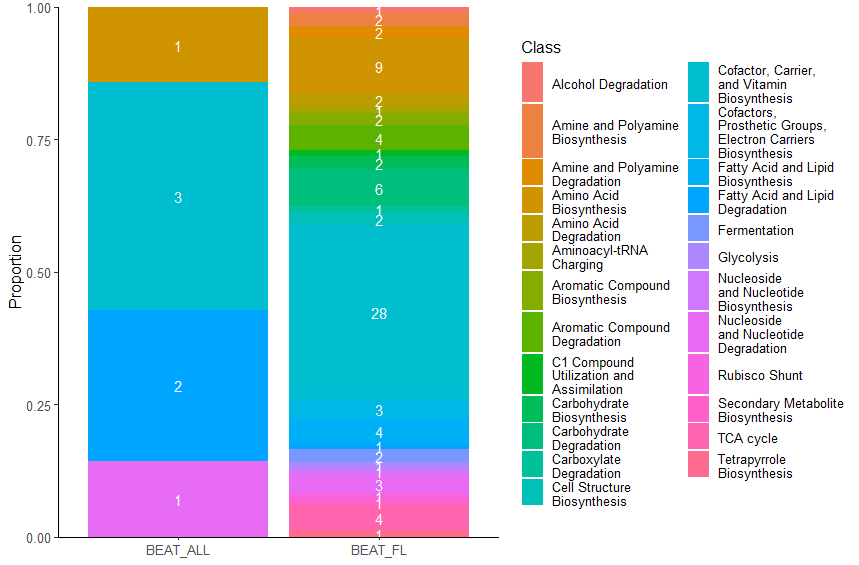

Supplement: S10 Fig — Association analysis between stratified pathway abundances and EEG. The analyzed pathways come from the top five contributing bacteria, found in Fig 4. Association at the Class level for the variables BEAT_ALL and BEAT_FL. (TIF) [file pone.0288689.s010.tif]
